# [*N*-(3-Meth­oxy-2-oxidobenzyl­idene-κ*O* ^2^)leucinato-κ^2^ *N*,*O*](1,10-phenanthroline-κ^2^ *N*,*N*′)copper(II) monohydrate

**DOI:** 10.1107/S160053681004554X

**Published:** 2010-11-13

**Authors:** Lei Huang, Jianfang Dong, Buqin Jing, Lianzhi Li, Daqi Wang

**Affiliations:** a School of Chemistry and Chemical Engineering, Liaocheng University, Shandong 252059, People’s Republic of China

## Abstract

The asymmetric unit of the title complex, [Cu(C_14_H_17_NO_4_)(C_12_H_8_N_2_)]·H_2_O, contains two independent Cu^II^ complex mol­ecules and two uncoordinated water mol­ecules. In each complex mol­ecule, the Cu atom is *O*,*N*,*O*′-chelated by the tridentate Schiff base ligand and *N*,*N*′-chelated by the 1,10-phenanthroline ligand in a distorted square-pyramidal geometry. The Cu—N bond distances in the apical directions are 2.298 (4) and 2.268 (4) Å. In the crystal, inter­molecular O—H⋯O and C—H⋯O hydrogen bonds together with C—H⋯π inter­actions result in a three-dimensional supra­molecular structure.

## Related literature

For related structures, see: Elena *et al.* (1995 ▶); Qiu *et al.* (2008 ▶). 
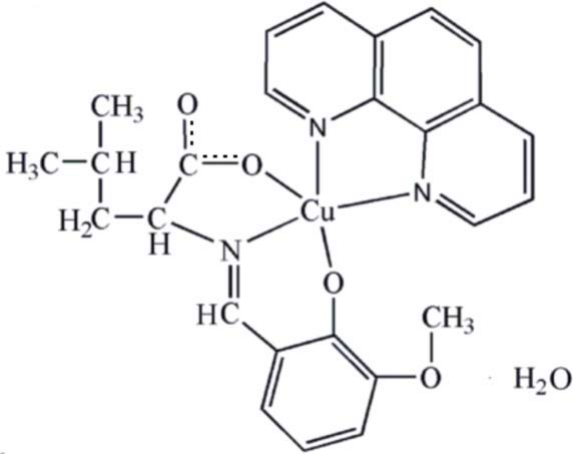


## Experimental

### 

#### Crystal data

[Cu(C_14_H_17_NO_4_)(C_12_H_8_N_2_)]·H_2_O
*M* *_r_* = 525.05
Monoclinic, 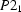

*a* = 11.1981 (12) Å
*b* = 10.4190 (11) Å
*c* = 21.298 (2) Å
β = 98.5520 (10)°
*V* = 2457.3 (5) Å^3^
*Z* = 4
Mo *K*α radiation
μ = 0.93 mm^−1^
*T* = 293 K
0.45 × 0.43 × 0.41 mm

#### Data collection

Bruker SMART 1000 CCD area-detector diffractometer
Absorption correction: multi-scan (*SADABS*; Sheldrick, 1996 ▶) *T*_min_ = 0.680, *T*_max_ = 0.702
12981 measured reflections
7382 independent reflections
5544 reflections with *I* > 2σ(*I*)
*R*_int_ = 0.027

#### Refinement

*R*[*F* ^2^ > 2σ(*F* ^2^)] = 0.038
*wR*(*F* ^2^) = 0.101
*S* = 1.01
7382 reflections
637 parameters
1 restraint
H-atom parameters constrained
Δρ_max_ = 0.42 e Å^−3^
Δρ_min_ = −0.30 e Å^−3^
Absolute structure: Flack (1983 ▶), 2779 Friedel pairs
Flack parameter: −0.010 (12)

### 

Data collection: *SMART* (Siemens, 1996 ▶); cell refinement: *SAINT* (Siemens, 1996 ▶); data reduction: *SAINT*; program(s) used to solve structure: *SHELXTL* (Sheldrick, 2008 ▶); program(s) used to refine structure: *SHELXTL*; molecular graphics: *SHELXTL*; software used to prepare material for publication: *SHELXTL*.

## Supplementary Material

Crystal structure: contains datablocks global, I. DOI: 10.1107/S160053681004554X/xu5077sup1.cif

Structure factors: contains datablocks I. DOI: 10.1107/S160053681004554X/xu5077Isup2.hkl

Additional supplementary materials:  crystallographic information; 3D view; checkCIF report

## Figures and Tables

**Table 1:** Selected bond lengths (Å)

Cu1—N1	1.919 (4)
Cu1—N2	2.010 (4)
Cu1—N3	2.298 (4)
Cu1—O1	1.960 (3)
Cu1—O3	1.929 (3)
Cu2—N4	1.934 (4)
Cu2—N5	2.268 (4)
Cu2—N6	2.039 (4)
Cu2—O5	1.953 (4)
Cu2—O7	1.945 (3)

**Table 2:** Hydrogen-bond geometry (Å, °)

*D*—H⋯*A*	*D*—H	H⋯*A*	*D*⋯*A*	*D*—H⋯*A*
O9—H9*A*⋯O2	0.85	2.22	2.715 (7)	117
O9—H9*B*⋯O2	0.85	2.20	2.715 (7)	119
O10—H10*A*⋯O6	0.85	2.27	2.805 (8)	121
O10—H10*B*⋯O6	0.85	2.27	2.805 (8)	121
C24—H24⋯O10^i^	0.93	2.58	3.476 (8)	162
C40—H40*A*⋯O2^ii^	0.96	2.53	3.486 (7)	176
C43—H43⋯O8^iii^	0.93	2.48	3.172 (6)	131
C50—H50⋯O9^iv^	0.93	2.35	3.268 (7)	168
C25—H25⋯*Cg*1^v^	0.93	2.51	3.426 (6)	168

Symmetry codes: (i) 
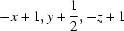
; (ii) 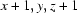; (iii) 
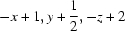
; (iv) 
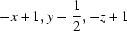
; (v) 
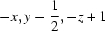
.

**Fig. 1.**
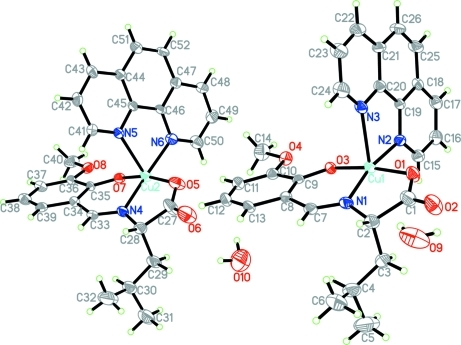
The structure of the title compound, showing 30% probability displacement ellipsoids and the atom-numbering scheme.

**Fig. 2.**
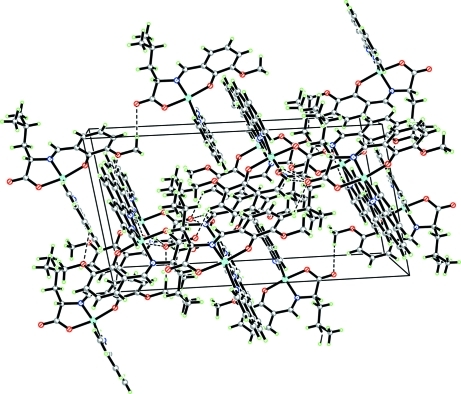
Packing diagram of the title compound.

## supplementary crystallographic information

### Comment

Schiff bases still play an important role as ligands in metal coordination chemistry even after almost a century since their discovery. Considerable efforts have been devotedd to copper(II) complexes of tridentate Schiff base ligands of *N*-alkylidene or *N*-arylidene alkanato type, due to their structural richness, electrochemical properties as well as a potential model for a number of important biological systems. Herein, we report the synthesis and crystal structure of a new copper(II) complex with a tridentate Schiff base ligand derived from the condensation of *o*-vanillin and *L*-leucine, and with 1,10-phenanthroline.

There are two independent structurses in the crystallographic asymmetric unit except the solvent water moleculars (Fig.1). In either of the two main molecular structures, the central Cu^II^ atom is five-coordinated by one N atom and two O atoms from the tridentate Schiff base ligand and two N atoms from 1,10-phenanthroline ligand, forming a seriously distorted square-pyramidal geomety. The O1, O3, N1, and N2 atoms (or O5, O7, N4, and N6) locate in equatorial plane, and N3 (or N5) in the apital position. The Cu1^II^ atom and Cu2^II^ atom lie 0.1552 (21)Å and 0.0859 (22)Å above theirs equatorial plane, respectively. Similar to that repoted previously (Elena *et al.*, 1995; Qiu *et al.*, 2008), the apital atoms (N3, N5) are much longer from their copper atoms, with the bond distances of Cu1—N3 = 2.298 (4)Å and Cu2—N5 = 2.268 (4)Å (Table 1). The tridentate Schiff base ligands coordinated to copper atoms form two penta-chelated rings(Cu1/O1/C1—C2/N1 and Cu2/O5/C27—C28/N4) and two hexa-chelated rings (Cu1/N1/C7—C9/O3 and Cu2/N4/C33—C35/O7). In each molecular, the penta-chelated ring and hexa-chelated ring have formed their individual dihedral angle 12.64 (22)° and 17.39 (21)°, respectively. The phenanthroline ligands are almost perpendicular to their individual equatorial plane (dihedral angle 89.03 (13)° and 86.24 (14)°).

In the crystal structure, there exist C—H···O and O—H···O hydrogen bonds (Table 2) which link the molecules into a three-dimensional network structure (Fig. 2). Additionally, the intermolecular C—H···π interaction [H25···*Cg*^i^= 2.513 Å, C25···*Cg*^i^= 3.426 Å, and C25—H25···*Cg*^i^= 167.55°; symmetry code: (i) -*x*, *y* - 1/2, 1 - *z*; *Cg* is the centroid of the C8—C13 ring] also stablizes the crystal packing, along with the van der Waals forces.

### Experimental

*L*-Leucine (1 mmol, 113.2 mg) and potassium hydroxide (1 mmol, 56.1 mg) were dissolved in hot methanol (10 ml) and added successively to a methanol solution of *o*-vanilline (1 mmol, 152.2 mg). The mixture was then stirred at 323 K for 2 h. Subsequently, an aqueous solution (2 ml) of cupric acetate monohydrate (1 mmol, 199.7 mg) was added dropwise and stirred for 2 h. An methanol solution (5 ml) of 1,10-phenanthroline monohydrate (1 mmol, 198.2 mg) was added dropwise and stirred for 4 h. The resultant solution was held at room temperature for 10 d, single crystals suitable for X-ray diffraction were obtained.

### Refinement

All the H atoms were placed in geometrically calculated positions with C—H = 0.93–0.98Å and O—H = 0.85 Å and allowed to ride on their parent atoms, with U_iso_(H) = 1.5U_eq_(C) for methyl H atoms and 1.2U_eq_(C) for the others.

### Crystal data

**Table:**

[Cu(C_14_H_17_NO_4_)(C_12_H_8_N_2_)]·H_2_O	*F*(000) = 1092
*M**_r_* = 525.05	*D*_x_ = 1.419 Mg m^−^^3^
Monoclinic, *P*2_1_	Mo *K*α radiation, λ = 0.71073 Å
Hall symbol: P 2yb	Cell parameters from 4089 reflections
*a* = 11.1981 (12) Å	θ = 2.2–26.8°
*b* = 10.4190 (11) Å	µ = 0.93 mm^−^^1^
*c* = 21.298 (2) Å	*T* = 293 K
β = 98.552 (1)°	Block, blue
*V* = 2457.3 (5) Å^3^	0.45 × 0.43 × 0.41 mm
*Z* = 4	

### Data collection

**Table:**

Bruker SMART 1000 CCD area-detector diffractometer	7382 independent reflections
Radiation source: fine-focus sealed tube	5544 reflections with *I* > 2σ(*I*)
graphite	*R*_int_ = 0.027
φ and ω scans	θ_max_ = 25.0°, θ_min_ = 1.9°
Absorption correction: multi-scan (*SADABS*; Sheldrick, 1996)	*h* = −13→11
*T*_min_ = 0.680, *T*_max_ = 0.702	*k* = −11→12
12981 measured reflections	*l* = −25→25

### Refinement

**Table:**

Refinement on *F*^2^	Secondary atom site location: difference Fourier map
Least-squares matrix: full	Hydrogen site location: inferred from neighbouring sites
*R*[*F*^2^ > 2σ(*F*^2^)] = 0.038	H-atom parameters constrained
*wR*(*F*^2^) = 0.101	*w* = 1/[σ^2^(*F*_o_^2^) + (0.0508*P*)^2^] where *P* = (*F*_o_^2^ + 2*F*_c_^2^)/3
*S* = 1.01	(Δ/σ)_max_ = 0.001
7382 reflections	Δρ_max_ = 0.42 e Å^−^^3^
637 parameters	Δρ_min_ = −0.30 e Å^−^^3^
1 restraint	Absolute structure: Flack (1983), 2779 Friedel pairs
Primary atom site location: structure-invariant direct methods	Flack parameter: −0.010 (12)

### Special details

**Table:**

Geometry. All e.s.d.'s (except the e.s.d. in the dihedral angle between two l.s. planes) are estimated using the full covariance matrix. The cell e.s.d.'s are taken into account individually in the estimation of e.s.d.'s in distances, angles and torsion angles; correlations between e.s.d.'s in cell parameters are only used when they are defined by crystal symmetry. An approximate (isotropic) treatment of cell e.s.d.'s is used for estimating e.s.d.'s involving l.s. planes.
Refinement. Refinement of *F*^2^ against ALL reflections. The weighted *R*-factor *wR* and goodness of fit *S* are based on *F*^2^, conventional *R*-factors *R* are based on *F*, with *F* set to zero for negative *F*^2^. The threshold expression of *F*^2^ > σ(*F*^2^) is used only for calculating *R*-factors(gt) *etc*. and is not relevant to the choice of reflections for refinement. *R*-factors based on *F*^2^ are statistically about twice as large as those based on *F*, and *R*- factors based on ALL data will be even larger.

### Fractional atomic coordinates and isotropic or equivalent isotropic displacement parameters (Å^2^)

**Table:**

	*x*	*y*	*z*	*U*_iso_*/*U*_eq_	
Cu1	0.19614 (5)	0.73977 (4)	0.38068 (3)	0.03585 (18)	
Cu2	0.69306 (5)	0.85315 (5)	0.88162 (3)	0.0400 (2)	
N1	0.3237 (3)	0.8364 (4)	0.35210 (16)	0.0402 (9)	
N2	0.0776 (3)	0.6083 (4)	0.40247 (18)	0.0336 (10)	
N3	0.0348 (3)	0.8640 (4)	0.3981 (2)	0.0388 (10)	
N4	0.8351 (3)	0.9294 (4)	0.85529 (17)	0.0426 (10)	
N5	0.5893 (3)	1.0317 (4)	0.90173 (19)	0.0359 (10)	
N6	0.5344 (4)	0.7771 (4)	0.9006 (2)	0.0427 (12)	
O1	0.1368 (3)	0.7246 (4)	0.28967 (15)	0.0522 (10)	
O2	0.1742 (4)	0.7864 (4)	0.19411 (19)	0.0757 (15)	
O3	0.2733 (3)	0.7420 (4)	0.46790 (14)	0.0348 (7)	
O4	0.3396 (3)	0.7325 (4)	0.59119 (15)	0.0429 (8)	
O5	0.6398 (3)	0.8405 (5)	0.79026 (17)	0.0636 (12)	
O6	0.6919 (4)	0.8878 (5)	0.69680 (19)	0.0940 (19)	
O7	0.7729 (3)	0.8381 (3)	0.96887 (15)	0.0365 (8)	
O8	0.8331 (3)	0.8287 (3)	1.09201 (15)	0.0452 (9)	
O9	0.2665 (4)	0.9682 (6)	0.1236 (2)	0.1102 (18)	
H9A	0.2637	0.9606	0.1631	0.132*	
H9B	0.2235	0.9014	0.1151	0.132*	
O10	0.7423 (5)	0.6893 (6)	0.6157 (3)	0.138 (2)	
H10A	0.7719	0.7625	0.6266	0.165*	
H10B	0.6913	0.7016	0.6410	0.165*	
C1	0.2027 (5)	0.7795 (6)	0.2530 (3)	0.0567 (16)	
C2	0.3243 (4)	0.8296 (6)	0.2836 (2)	0.0488 (13)	
H2	0.3389	0.9148	0.2669	0.059*	
C3	0.4194 (5)	0.7354 (7)	0.2679 (2)	0.0638 (17)	
H3A	0.4072	0.7226	0.2223	0.077*	
H3B	0.4054	0.6536	0.2872	0.077*	
C4	0.5513 (5)	0.7724 (7)	0.2886 (3)	0.074 (2)	
H4	0.5634	0.7897	0.3344	0.089*	
C5	0.6332 (6)	0.6615 (9)	0.2758 (3)	0.113 (3)	
H5A	0.6284	0.6492	0.2308	0.170*	
H5B	0.6078	0.5846	0.2948	0.170*	
H5C	0.7150	0.6809	0.2937	0.170*	
C6	0.5864 (6)	0.8914 (8)	0.2545 (4)	0.104 (3)	
H6A	0.6691	0.9128	0.2697	0.156*	
H6B	0.5353	0.9616	0.2626	0.156*	
H6C	0.5771	0.8750	0.2097	0.156*	
C7	0.3970 (4)	0.9105 (4)	0.3880 (2)	0.0404 (11)	
H7	0.4420	0.9684	0.3678	0.048*	
C8	0.4153 (4)	0.9117 (4)	0.4557 (2)	0.0348 (10)	
C9	0.3581 (4)	0.8213 (4)	0.4918 (2)	0.0320 (10)	
C10	0.3961 (4)	0.8229 (4)	0.5585 (2)	0.0370 (11)	
C11	0.4807 (4)	0.9088 (5)	0.5865 (2)	0.0449 (12)	
H11	0.5035	0.9078	0.6303	0.054*	
C12	0.5327 (4)	0.9974 (5)	0.5497 (2)	0.0473 (13)	
H12	0.5887	1.0565	0.5689	0.057*	
C13	0.5018 (4)	0.9973 (5)	0.4866 (3)	0.0430 (14)	
H13	0.5386	1.0556	0.4625	0.052*	
C14	0.3722 (5)	0.7327 (7)	0.6588 (2)	0.0672 (17)	
H14A	0.4585	0.7285	0.6694	0.101*	
H14B	0.3367	0.6597	0.6764	0.101*	
H14C	0.3432	0.8100	0.6759	0.101*	
C15	0.0984 (4)	0.4836 (5)	0.4038 (2)	0.0433 (13)	
H15	0.1702	0.4536	0.3921	0.052*	
C16	0.0177 (5)	0.3962 (5)	0.4217 (3)	0.0496 (14)	
H16	0.0361	0.3091	0.4220	0.060*	
C17	−0.0884 (5)	0.4358 (5)	0.4388 (3)	0.0486 (14)	
H17	−0.1425	0.3767	0.4513	0.058*	
C18	−0.1149 (4)	0.5669 (4)	0.4373 (2)	0.0348 (11)	
C19	−0.0288 (4)	0.6520 (4)	0.4180 (2)	0.0301 (11)	
C20	−0.0515 (4)	0.7856 (5)	0.4158 (2)	0.0358 (12)	
C21	−0.1618 (4)	0.8341 (5)	0.4321 (2)	0.0410 (12)	
C22	−0.1788 (5)	0.9677 (5)	0.4294 (3)	0.0559 (16)	
H22	−0.2489	1.0037	0.4403	0.067*	
C23	−0.0921 (5)	1.0440 (5)	0.4107 (3)	0.0606 (17)	
H23	−0.1033	1.1324	0.4083	0.073*	
C24	0.0125 (5)	0.9904 (5)	0.3953 (3)	0.0566 (16)	
H24	0.0702	1.0446	0.3823	0.068*	
C25	−0.2228 (4)	0.6194 (6)	0.4546 (2)	0.0492 (14)	
H25	−0.2793	0.5644	0.4679	0.059*	
C26	−0.2453 (4)	0.7458 (6)	0.4521 (2)	0.0486 (13)	
H26	−0.3169	0.7763	0.4637	0.058*	
C27	0.7124 (5)	0.8792 (7)	0.7547 (3)	0.0681 (19)	
C28	0.8379 (4)	0.9240 (6)	0.7871 (2)	0.0506 (13)	
H28	0.8577	1.0083	0.7711	0.061*	
C29	0.9290 (5)	0.8203 (6)	0.7707 (3)	0.0612 (18)	
H29A	0.9089	0.7395	0.7893	0.073*	
H29B	0.9169	0.8090	0.7250	0.073*	
C30	1.0599 (5)	0.8464 (7)	0.7920 (3)	0.0636 (17)	
H30	1.0732	0.8544	0.8384	0.076*	
C31	1.1342 (6)	0.7372 (9)	0.7741 (3)	0.094 (2)	
H31A	1.1273	0.7323	0.7287	0.141*	
H31B	1.1059	0.6586	0.7902	0.141*	
H31C	1.2172	0.7505	0.7919	0.141*	
C32	1.1026 (6)	0.9700 (8)	0.7637 (4)	0.100 (3)	
H32A	1.1055	0.9577	0.7193	0.150*	
H32B	1.1817	0.9918	0.7850	0.150*	
H32C	1.0474	1.0383	0.7691	0.150*	
C33	0.9021 (4)	1.0065 (5)	0.8911 (2)	0.0412 (11)	
H33	0.9494	1.0630	0.8717	0.049*	
C34	0.9109 (4)	1.0140 (4)	0.9588 (2)	0.0368 (11)	
C35	0.8521 (3)	0.9214 (4)	0.9934 (2)	0.0326 (10)	
C36	0.8865 (3)	0.9238 (4)	1.0618 (2)	0.0332 (10)	
C37	0.9640 (4)	1.0140 (5)	1.0907 (2)	0.0430 (12)	
H37	0.9846	1.0128	1.1346	0.052*	
C38	1.0132 (4)	1.1088 (5)	1.0552 (3)	0.0480 (13)	
H38	1.0622	1.1730	1.0756	0.058*	
C39	0.9887 (4)	1.1058 (5)	0.9915 (3)	0.0434 (13)	
H39	1.0245	1.1665	0.9682	0.052*	
C40	0.8619 (5)	0.8225 (6)	1.1594 (2)	0.0650 (18)	
H40A	0.9475	0.8123	1.1711	0.097*	
H40B	0.8211	0.7507	1.1749	0.097*	
H40C	0.8365	0.9003	1.1777	0.097*	
C41	0.6150 (5)	1.1534 (5)	0.9030 (2)	0.0462 (14)	
H41	0.6885	1.1782	0.8914	0.055*	
C42	0.5391 (4)	1.2499 (5)	0.9208 (2)	0.0451 (13)	
H42	0.5613	1.3359	0.9206	0.054*	
C43	0.4313 (4)	1.2134 (5)	0.9384 (2)	0.0476 (14)	
H43	0.3795	1.2749	0.9509	0.057*	
C44	0.3996 (4)	1.0850 (5)	0.9375 (2)	0.0378 (12)	
C45	0.4820 (4)	0.9955 (5)	0.9187 (2)	0.0317 (11)	
C46	0.4529 (4)	0.8625 (5)	0.9170 (2)	0.0357 (12)	
C47	0.3416 (4)	0.8224 (5)	0.9327 (2)	0.0417 (13)	
C48	0.3171 (5)	0.6905 (6)	0.9303 (3)	0.0559 (16)	
H48	0.2445	0.6600	0.9407	0.067*	
C49	0.3979 (5)	0.6082 (5)	0.9129 (3)	0.0568 (15)	
H49	0.3808	0.5208	0.9105	0.068*	
C50	0.5081 (5)	0.6536 (5)	0.8984 (3)	0.0539 (16)	
H50	0.5641	0.5955	0.8870	0.065*	
C51	0.2882 (4)	1.0414 (5)	0.9544 (2)	0.0494 (14)	
H51	0.2347	1.1003	0.9676	0.059*	
C52	0.2598 (4)	0.9170 (6)	0.9517 (3)	0.0487 (15)	
H52	0.1859	0.8907	0.9622	0.058*	

### Atomic displacement parameters (Å^2^)

**Table:**

	*U*^11^	*U*^22^	*U*^33^	*U*^12^	*U*^13^	*U*^23^
Cu1	0.0293 (4)	0.0392 (4)	0.0409 (4)	−0.0066 (3)	0.0113 (3)	−0.0029 (3)
Cu2	0.0304 (4)	0.0484 (4)	0.0421 (4)	0.0046 (3)	0.0083 (3)	−0.0029 (3)
N1	0.034 (2)	0.052 (3)	0.038 (2)	−0.0060 (19)	0.0152 (17)	0.000 (2)
N2	0.032 (2)	0.033 (2)	0.038 (2)	−0.0035 (18)	0.0112 (17)	−0.0033 (19)
N3	0.031 (2)	0.033 (2)	0.053 (3)	−0.009 (2)	0.0092 (19)	0.000 (2)
N4	0.032 (2)	0.062 (3)	0.035 (2)	0.009 (2)	0.0097 (17)	0.008 (2)
N5	0.031 (2)	0.034 (3)	0.044 (3)	0.0055 (18)	0.0093 (18)	0.002 (2)
N6	0.037 (3)	0.043 (3)	0.048 (3)	−0.0005 (19)	0.008 (2)	−0.0031 (19)
O1	0.044 (2)	0.071 (3)	0.042 (2)	−0.021 (2)	0.0089 (16)	−0.004 (2)
O2	0.063 (3)	0.124 (4)	0.038 (2)	−0.022 (2)	0.0001 (19)	0.011 (2)
O3	0.0316 (17)	0.0334 (17)	0.0404 (19)	−0.0082 (16)	0.0088 (14)	−0.0032 (18)
O4	0.0490 (19)	0.0399 (19)	0.040 (2)	−0.0017 (17)	0.0069 (15)	0.0037 (18)
O5	0.038 (2)	0.107 (4)	0.046 (2)	−0.001 (2)	0.0070 (18)	−0.005 (2)
O6	0.064 (3)	0.177 (6)	0.039 (2)	0.017 (3)	0.004 (2)	0.007 (3)
O7	0.0317 (18)	0.036 (2)	0.042 (2)	−0.0040 (16)	0.0067 (15)	0.0003 (17)
O8	0.0455 (19)	0.050 (2)	0.041 (2)	−0.0049 (17)	0.0096 (16)	0.0081 (17)
O9	0.067 (3)	0.128 (5)	0.139 (4)	0.008 (3)	0.027 (3)	0.050 (4)
O10	0.139 (5)	0.098 (4)	0.176 (6)	0.000 (4)	0.024 (4)	−0.028 (4)
C1	0.043 (3)	0.076 (5)	0.052 (4)	−0.007 (3)	0.008 (3)	−0.002 (3)
C2	0.044 (3)	0.065 (4)	0.041 (3)	−0.012 (3)	0.018 (2)	0.000 (3)
C3	0.055 (3)	0.095 (5)	0.045 (3)	−0.010 (4)	0.020 (3)	−0.019 (4)
C4	0.053 (4)	0.121 (7)	0.053 (4)	0.001 (4)	0.021 (3)	−0.015 (4)
C5	0.084 (5)	0.166 (10)	0.098 (6)	0.035 (6)	0.039 (4)	−0.003 (6)
C6	0.066 (5)	0.138 (8)	0.116 (6)	−0.029 (5)	0.040 (4)	−0.023 (5)
C7	0.037 (3)	0.036 (3)	0.053 (3)	−0.008 (2)	0.022 (2)	−0.002 (2)
C8	0.030 (2)	0.030 (2)	0.046 (3)	−0.0021 (19)	0.013 (2)	−0.005 (2)
C9	0.027 (2)	0.027 (3)	0.045 (3)	0.0041 (18)	0.0135 (19)	−0.004 (2)
C10	0.033 (2)	0.033 (3)	0.045 (3)	0.004 (2)	0.007 (2)	0.000 (2)
C11	0.036 (3)	0.046 (3)	0.051 (3)	0.000 (2)	0.002 (2)	−0.007 (2)
C12	0.033 (3)	0.041 (3)	0.065 (4)	−0.007 (2)	0.001 (2)	−0.011 (3)
C13	0.027 (2)	0.038 (3)	0.066 (4)	−0.009 (2)	0.012 (3)	−0.008 (3)
C14	0.083 (4)	0.073 (4)	0.047 (4)	−0.007 (4)	0.015 (3)	0.007 (3)
C15	0.039 (3)	0.039 (3)	0.053 (3)	−0.003 (2)	0.010 (2)	−0.005 (3)
C16	0.054 (3)	0.026 (3)	0.070 (4)	−0.009 (2)	0.012 (3)	−0.004 (2)
C17	0.042 (3)	0.044 (4)	0.060 (4)	−0.013 (3)	0.012 (3)	−0.001 (3)
C18	0.029 (2)	0.034 (3)	0.041 (3)	−0.006 (2)	0.003 (2)	0.001 (2)
C19	0.026 (2)	0.028 (3)	0.036 (3)	−0.004 (2)	0.006 (2)	−0.003 (2)
C20	0.034 (3)	0.036 (3)	0.038 (3)	−0.002 (2)	0.007 (2)	0.000 (2)
C21	0.033 (3)	0.037 (3)	0.055 (3)	0.006 (2)	0.012 (2)	−0.006 (3)
C22	0.042 (3)	0.047 (4)	0.078 (4)	0.012 (3)	0.006 (3)	−0.005 (3)
C23	0.049 (3)	0.029 (3)	0.104 (5)	0.005 (3)	0.011 (3)	0.007 (3)
C24	0.053 (4)	0.035 (3)	0.083 (4)	−0.006 (3)	0.014 (3)	0.008 (3)
C25	0.034 (3)	0.051 (4)	0.067 (4)	−0.010 (3)	0.022 (2)	0.002 (3)
C26	0.033 (3)	0.054 (4)	0.063 (3)	0.005 (3)	0.018 (2)	0.002 (3)
C27	0.048 (3)	0.108 (6)	0.046 (4)	0.028 (3)	−0.002 (3)	−0.008 (3)
C28	0.044 (3)	0.070 (4)	0.039 (3)	0.006 (3)	0.012 (2)	0.009 (3)
C29	0.045 (3)	0.094 (5)	0.049 (3)	0.017 (3)	0.021 (3)	0.006 (3)
C30	0.045 (3)	0.102 (5)	0.045 (4)	0.018 (4)	0.014 (3)	0.012 (4)
C31	0.064 (4)	0.133 (6)	0.091 (5)	0.034 (5)	0.031 (4)	0.016 (5)
C32	0.061 (4)	0.108 (6)	0.139 (7)	−0.007 (4)	0.039 (4)	0.004 (6)
C33	0.029 (2)	0.048 (3)	0.050 (3)	0.006 (2)	0.017 (2)	0.010 (2)
C34	0.029 (2)	0.036 (3)	0.046 (3)	0.003 (2)	0.0073 (19)	0.004 (2)
C35	0.027 (2)	0.027 (2)	0.046 (3)	0.0077 (19)	0.0124 (19)	0.004 (2)
C36	0.026 (2)	0.030 (3)	0.044 (3)	0.0000 (19)	0.0057 (19)	0.004 (2)
C37	0.034 (2)	0.050 (3)	0.045 (3)	0.000 (2)	0.007 (2)	−0.009 (2)
C38	0.036 (3)	0.040 (3)	0.068 (4)	−0.008 (2)	0.005 (2)	−0.011 (3)
C39	0.034 (3)	0.038 (3)	0.061 (4)	−0.007 (2)	0.015 (3)	0.004 (3)
C40	0.065 (4)	0.090 (5)	0.041 (3)	−0.016 (3)	0.013 (3)	0.006 (3)
C41	0.040 (3)	0.046 (4)	0.054 (3)	0.004 (2)	0.009 (2)	0.014 (3)
C42	0.047 (3)	0.034 (3)	0.052 (3)	0.007 (2)	0.000 (2)	0.001 (3)
C43	0.039 (3)	0.047 (4)	0.058 (3)	0.015 (2)	0.011 (2)	0.004 (3)
C44	0.032 (3)	0.040 (3)	0.042 (3)	0.003 (2)	0.007 (2)	0.000 (3)
C45	0.026 (2)	0.038 (3)	0.031 (3)	0.003 (2)	0.0031 (19)	0.002 (2)
C46	0.022 (2)	0.049 (3)	0.035 (3)	0.000 (2)	0.0030 (19)	−0.001 (3)
C47	0.036 (3)	0.040 (3)	0.048 (3)	−0.002 (2)	0.002 (2)	0.002 (3)
C48	0.035 (3)	0.064 (4)	0.067 (4)	−0.005 (3)	0.003 (3)	0.012 (3)
C49	0.057 (4)	0.029 (3)	0.080 (4)	−0.009 (3)	−0.002 (3)	−0.001 (3)
C50	0.048 (3)	0.044 (4)	0.069 (4)	0.002 (3)	0.006 (3)	−0.011 (3)
C51	0.037 (3)	0.052 (4)	0.061 (4)	0.010 (3)	0.015 (2)	0.001 (3)
C52	0.023 (3)	0.068 (4)	0.056 (4)	0.000 (3)	0.011 (2)	0.008 (3)

### Geometric parameters (Å, °)

**Table:**

Cu1—N1	1.919 (4)	C16—C17	1.358 (7)
Cu1—N2	2.010 (4)	C16—H16	0.9300
Cu1—N3	2.298 (4)	C17—C18	1.398 (7)
Cu1—O1	1.960 (3)	C17—H17	0.9300
Cu1—O3	1.929 (3)	C18—C19	1.414 (6)
Cu2—N4	1.934 (4)	C18—C25	1.424 (7)
Cu2—N5	2.268 (4)	C19—C20	1.415 (6)
Cu2—N6	2.039 (4)	C20—C21	1.425 (6)
Cu2—O5	1.953 (4)	C21—C22	1.405 (7)
Cu2—O7	1.945 (3)	C21—C26	1.421 (7)
N1—C7	1.291 (5)	C22—C23	1.360 (7)
N1—C2	1.461 (5)	C22—H22	0.9300
N2—C15	1.319 (6)	C23—C24	1.380 (7)
N2—C19	1.361 (6)	C23—H23	0.9300
N3—C24	1.341 (7)	C24—H24	0.9300
N3—C20	1.360 (6)	C25—C26	1.340 (7)
N4—C33	1.273 (6)	C25—H25	0.9300
N4—C28	1.458 (6)	C26—H26	0.9300
N5—C41	1.300 (6)	C27—C28	1.543 (7)
N5—C45	1.359 (5)	C28—C29	1.560 (7)
N6—C50	1.319 (7)	C28—H28	0.9800
N6—C46	1.357 (6)	C29—C30	1.494 (7)
O1—C1	1.286 (6)	C29—H29A	0.9700
O2—C1	1.249 (6)	C29—H29B	0.9700
O3—C9	1.303 (5)	C30—C31	1.492 (9)
O4—C10	1.379 (5)	C30—C32	1.529 (10)
O4—C14	1.431 (5)	C30—H30	0.9800
O5—C27	1.258 (7)	C31—H31A	0.9600
O6—C27	1.223 (6)	C31—H31B	0.9600
O7—C35	1.293 (5)	C31—H31C	0.9600
O8—C36	1.366 (5)	C32—H32A	0.9600
O8—C40	1.426 (6)	C32—H32B	0.9600
O9—H9A	0.8501	C32—H32C	0.9600
O9—H9B	0.8500	C33—C34	1.432 (6)
O10—H10A	0.8500	C33—H33	0.9300
O10—H10B	0.8500	C34—C39	1.406 (6)
C1—C2	1.512 (7)	C34—C35	1.433 (6)
C2—C3	1.521 (8)	C35—C36	1.449 (6)
C2—H2	0.9800	C36—C37	1.363 (6)
C3—C4	1.527 (7)	C37—C38	1.405 (7)
C3—H3A	0.9700	C37—H37	0.9300
C3—H3B	0.9700	C38—C39	1.346 (7)
C4—C6	1.518 (10)	C38—H38	0.9300
C4—C5	1.525 (9)	C39—H39	0.9300
C4—H4	0.9800	C40—H40A	0.9600
C5—H5A	0.9600	C40—H40B	0.9600
C5—H5B	0.9600	C40—H40C	0.9600
C5—H5C	0.9600	C41—C42	1.405 (7)
C6—H6A	0.9600	C41—H41	0.9300
C6—H6B	0.9600	C42—C43	1.370 (7)
C6—H6C	0.9600	C42—H42	0.9300
C7—C8	1.426 (6)	C43—C44	1.384 (7)
C7—H7	0.9300	C43—H43	0.9300
C8—C13	1.406 (6)	C44—C45	1.411 (6)
C8—C9	1.427 (6)	C44—C51	1.423 (7)
C9—C10	1.422 (6)	C45—C46	1.423 (7)
C10—C11	1.373 (6)	C46—C47	1.400 (6)
C11—C12	1.394 (7)	C47—C48	1.401 (8)
C11—H11	0.9300	C47—C52	1.444 (7)
C12—C13	1.336 (7)	C48—C49	1.338 (7)
C12—H12	0.9300	C48—H48	0.9300
C13—H13	0.9300	C49—C50	1.398 (8)
C14—H14A	0.9600	C49—H49	0.9300
C14—H14B	0.9600	C50—H50	0.9300
C14—H14C	0.9600	C51—C52	1.334 (7)
C15—C16	1.376 (7)	C51—H51	0.9300
C15—H15	0.9300	C52—H52	0.9300
			
N1—Cu1—O3	92.92 (14)	N2—C19—C20	118.7 (4)
N1—Cu1—O1	83.54 (15)	C18—C19—C20	120.0 (4)
O3—Cu1—O1	172.26 (15)	N3—C20—C19	118.0 (4)
N1—Cu1—N2	168.34 (16)	N3—C20—C21	122.1 (5)
O3—Cu1—N2	90.15 (15)	C19—C20—C21	119.9 (4)
O1—Cu1—N2	92.01 (16)	C22—C21—C26	124.2 (5)
N1—Cu1—N3	113.34 (16)	C22—C21—C20	117.3 (5)
O3—Cu1—N3	95.04 (14)	C26—C21—C20	118.5 (5)
O1—Cu1—N3	92.68 (15)	C23—C22—C21	119.5 (5)
N2—Cu1—N3	77.54 (14)	C23—C22—H22	120.2
N4—Cu2—O7	91.66 (14)	C21—C22—H22	120.2
N4—Cu2—O5	83.18 (16)	C22—C23—C24	120.1 (5)
O7—Cu2—O5	167.25 (17)	C22—C23—H23	119.9
N4—Cu2—N6	174.25 (17)	C24—C23—H23	119.9
O7—Cu2—N6	94.02 (16)	N3—C24—C23	123.0 (5)
O5—Cu2—N6	91.43 (18)	N3—C24—H24	118.5
N4—Cu2—N5	100.62 (15)	C23—C24—H24	118.5
O7—Cu2—N5	93.51 (15)	C26—C25—C18	121.9 (5)
O5—Cu2—N5	98.88 (17)	C26—C25—H25	119.0
N6—Cu2—N5	78.18 (15)	C18—C25—H25	119.0
C7—N1—C2	121.6 (4)	C25—C26—C21	121.3 (5)
C7—N1—Cu1	124.3 (3)	C25—C26—H26	119.4
C2—N1—Cu1	113.9 (3)	C21—C26—H26	119.4
C15—N2—C19	118.8 (4)	O6—C27—O5	126.1 (6)
C15—N2—Cu1	123.8 (3)	O6—C27—C28	116.9 (6)
C19—N2—Cu1	117.4 (3)	O5—C27—C28	117.0 (5)
C24—N3—C20	117.9 (4)	N4—C28—C27	107.7 (4)
C24—N3—Cu1	133.8 (4)	N4—C28—C29	111.2 (4)
C20—N3—Cu1	108.3 (3)	C27—C28—C29	105.8 (5)
C33—N4—C28	121.6 (4)	N4—C28—H28	110.6
C33—N4—Cu2	121.5 (3)	C27—C28—H28	110.6
C28—N4—Cu2	114.4 (3)	C29—C28—H28	110.6
C41—N5—C45	117.9 (4)	C30—C29—C28	116.8 (5)
C41—N5—Cu2	133.3 (4)	C30—C29—H29A	108.1
C45—N5—Cu2	108.7 (3)	C28—C29—H29A	108.1
C50—N6—C46	119.5 (5)	C30—C29—H29B	108.1
C50—N6—Cu2	124.7 (4)	C28—C29—H29B	108.1
C46—N6—Cu2	115.8 (3)	H29A—C29—H29B	107.3
C1—O1—Cu1	115.1 (3)	C31—C30—C29	110.1 (6)
C9—O3—Cu1	125.4 (3)	C31—C30—C32	108.9 (5)
C10—O4—C14	116.0 (4)	C29—C30—C32	112.6 (5)
C27—O5—Cu2	116.7 (4)	C31—C30—H30	108.4
C35—O7—Cu2	121.5 (3)	C29—C30—H30	108.4
C36—O8—C40	117.3 (4)	C32—C30—H30	108.4
H9A—O9—H9B	91.9	C30—C31—H31A	109.5
H10A—O10—H10B	88.0	C30—C31—H31B	109.5
O2—C1—O1	123.4 (5)	H31A—C31—H31B	109.5
O2—C1—C2	119.5 (5)	C30—C31—H31C	109.5
O1—C1—C2	116.9 (5)	H31A—C31—H31C	109.5
N1—C2—C1	108.1 (4)	H31B—C31—H31C	109.5
N1—C2—C3	111.0 (4)	C30—C32—H32A	109.5
C1—C2—C3	107.3 (4)	C30—C32—H32B	109.5
N1—C2—H2	110.1	H32A—C32—H32B	109.5
C1—C2—H2	110.1	C30—C32—H32C	109.5
C3—C2—H2	110.1	H32A—C32—H32C	109.5
C2—C3—C4	116.9 (5)	H32B—C32—H32C	109.5
C2—C3—H3A	108.1	N4—C33—C34	125.7 (4)
C4—C3—H3A	108.1	N4—C33—H33	117.1
C2—C3—H3B	108.1	C34—C33—H33	117.1
C4—C3—H3B	108.1	C39—C34—C33	118.7 (4)
H3A—C3—H3B	107.3	C39—C34—C35	120.1 (4)
C6—C4—C5	109.1 (6)	C33—C34—C35	120.9 (4)
C6—C4—C3	112.1 (6)	O7—C35—C34	125.7 (4)
C5—C4—C3	109.8 (6)	O7—C35—C36	118.8 (4)
C6—C4—H4	108.6	C34—C35—C36	115.5 (4)
C5—C4—H4	108.6	C37—C36—O8	125.5 (4)
C3—C4—H4	108.6	C37—C36—C35	121.5 (4)
C4—C5—H5A	109.5	O8—C36—C35	113.0 (4)
C4—C5—H5B	109.5	C36—C37—C38	121.1 (4)
H5A—C5—H5B	109.5	C36—C37—H37	119.4
C4—C5—H5C	109.5	C38—C37—H37	119.4
H5A—C5—H5C	109.5	C39—C38—C37	119.2 (5)
H5B—C5—H5C	109.5	C39—C38—H38	120.4
C4—C6—H6A	109.5	C37—C38—H38	120.4
C4—C6—H6B	109.5	C38—C39—C34	122.2 (5)
H6A—C6—H6B	109.5	C38—C39—H39	118.9
C4—C6—H6C	109.5	C34—C39—H39	118.9
H6A—C6—H6C	109.5	O8—C40—H40A	109.5
H6B—C6—H6C	109.5	O8—C40—H40B	109.5
N1—C7—C8	125.9 (4)	H40A—C40—H40B	109.5
N1—C7—H7	117.0	O8—C40—H40C	109.5
C8—C7—H7	117.0	H40A—C40—H40C	109.5
C13—C8—C7	117.7 (4)	H40B—C40—H40C	109.5
C13—C8—C9	120.0 (4)	N5—C41—C42	124.3 (5)
C7—C8—C9	122.0 (4)	N5—C41—H41	117.9
O3—C9—C10	119.4 (4)	C42—C41—H41	117.9
O3—C9—C8	124.6 (4)	C43—C42—C41	117.9 (5)
C10—C9—C8	116.0 (4)	C43—C42—H42	121.0
C11—C10—O4	124.4 (4)	C41—C42—H42	121.0
C11—C10—C9	121.7 (4)	C42—C43—C44	119.9 (5)
O4—C10—C9	113.8 (4)	C42—C43—H43	120.0
C10—C11—C12	120.5 (4)	C44—C43—H43	120.0
C10—C11—H11	119.8	C43—C44—C45	117.8 (4)
C12—C11—H11	119.8	C43—C44—C51	122.5 (5)
C13—C12—C11	119.8 (5)	C45—C44—C51	119.7 (5)
C13—C12—H12	120.1	N5—C45—C44	122.2 (4)
C11—C12—H12	120.1	N5—C45—C46	118.2 (4)
C12—C13—C8	121.9 (5)	C44—C45—C46	119.6 (4)
C12—C13—H13	119.0	N6—C46—C47	121.5 (5)
C8—C13—H13	119.0	N6—C46—C45	119.0 (4)
O4—C14—H14A	109.5	C47—C46—C45	119.5 (4)
O4—C14—H14B	109.5	C46—C47—C48	117.4 (5)
H14A—C14—H14B	109.5	C46—C47—C52	119.1 (5)
O4—C14—H14C	109.5	C48—C47—C52	123.4 (5)
H14A—C14—H14C	109.5	C49—C48—C47	120.1 (5)
H14B—C14—H14C	109.5	C49—C48—H48	120.0
N2—C15—C16	122.4 (5)	C47—C48—H48	120.0
N2—C15—H15	118.8	C48—C49—C50	120.0 (5)
C16—C15—H15	118.8	C48—C49—H49	120.0
C17—C16—C15	120.7 (5)	C50—C49—H49	120.0
C17—C16—H16	119.7	N6—C50—C49	121.5 (5)
C15—C16—H16	119.7	N6—C50—H50	119.2
C16—C17—C18	118.8 (5)	C49—C50—H50	119.2
C16—C17—H17	120.6	C52—C51—C44	120.7 (5)
C18—C17—H17	120.6	C52—C51—H51	119.6
C17—C18—C19	117.9 (4)	C44—C51—H51	119.6
C17—C18—C25	123.7 (5)	C51—C52—C47	121.3 (5)
C19—C18—C25	118.4 (4)	C51—C52—H52	119.4
N2—C19—C18	121.3 (4)	C47—C52—H52	119.4
			
C1—C2—C3—C4	174.4 (5)		

### Hydrogen-bond geometry (Å, °)

**Table:**

Cg1 is the centroid of the C8–C13 ring.

**Table:**

*D*—H···*A*	*D*—H	H···*A*	*D*···*A*	*D*—H···*A*
O9—H9A···O2	0.85	2.22	2.715 (7)	117
O9—H9B···O2	0.85	2.20	2.715 (7)	119
O10—H10A···O6	0.85	2.27	2.805 (8)	121
O10—H10B···O6	0.85	2.27	2.805 (8)	121
C24—H24···O10^i^	0.93	2.58	3.476 (8)	162
C40—H40A···O2^ii^	0.96	2.53	3.486 (7)	176
C43—H43···O8^iii^	0.93	2.48	3.172 (6)	131
C50—H50···O9^iv^	0.93	2.35	3.268 (7)	168
C25—H25···Cg1^v^	0.93	2.51	3.426 (6)	168

Symmetry codes: (i) −*x*+1, *y*+1/2, −*z*+1; (ii) *x*+1, *y*, *z*+1; (iii) −*x*+1, *y*+1/2, −*z*+2; (iv) −*x*+1, *y*−1/2, −*z*+1; (v) −*x*, *y*−1/2, −*z*+1.
